# Beyond risk reduction of work-related musculoskeletal disorders: The CoWork musculoskeletal health model

**DOI:** 10.5271/sjweh.4262

**Published:** 2026-05-01

**Authors:** Andreas Holtermann, Ole H Sørensen, Sandra S Jacobsen, Line Lindberg, Lars L Andersen

**Affiliations:** 1 National Research Centre for the Working Environment, Copenhagen, Denmark.

**Keywords:** conceptual model, MSD, MSH, musculoskeletal disease, psychosocial work factor, physical work factor, workplace prevention

## Abstract

**Objectives:**

Work-related musculoskeletal disorders (MSD) affect over 1.7 billion people globally with a huge economic burden. Despite decades with legislations, policies and risk-reduction interventions, we see no decreases in MSD prevalence. Current prevention models focus on eliminating workplace hazards, overlooking that physical and psychosocial work factors can also promote musculoskeletal health (MSH). We were commissioned through the Danish Working Environment Agreement to develop new approaches addressing this conceptual gap.

**Methods:**

Through iterative stakeholder dialogue with Danish policymakers, social partners, and workplace practitioners, we developed and visualized the CoWork (Copenhagen Work-related) MSH model to shift the focus from preventing MSD through risk reduction toward actively promoting work-related MSH. The model aims to bridge theory with workplace structure and implementation by addressing stakeholder requests for clear terminology, conceptual understanding in a workplace context, and actionable guidance.

**Results:**

The CoWork MSH model provides a new definition of work-related MSH as “a state of physical, mental, and social well-being of the locomotor system in relation to work” as well as five integrated elements; (i) a health-oriented approach, (ii) a just-right work factor conceptualization, (iii) the Organizational, Management, Group, !ndividual (OMG!) workplace framework, (iv) an intervention guidance, and (v) health economics perspective. This approach recognizes that work factors can benefit rather than harm health when properly designed and implemented.

**Conclusions:**

The CoWork MSH model represents a paradigm shift, extending from risk reduction to MSH promotion, providing researchers, policymakers, and practitioners with a framework for understanding, researching, and practice to promote workplace MSH.

Work-related musculoskeletal disorders (MSD) represent the highest global health-related burden, affecting over 1.7 billion people worldwide and imposing a $2.1 trillion economic burden (1, 2). Despite five decades of research, policy initiatives, and workplace interventions focused on reducing physical and psychosocial risk factors, MSD prevalence continues to rise globally (3). Implementation failure of evidence-based workplace interventions on MSD might partly explain this. However, the global increase in MSD signals a fundamental limitation in our conceptual approach (theory), necessitating a paradigm shift from risk reduction to health promotion in occupational musculoskeletal health (MSH) (4, 5).

## Evolution of conceptual models

The conceptual understanding of work-related MSD has evolved over time. In the 1980s, influential models focused on biomechanical work factors such as high force, awkward postures, and repetitive work as causes to MSD (6, 7). In this period, models for acceptable whole-body energetic workloads (eg, based on heart rate monitoring) were also developed (8).

The entry of computer work in the early 1990s initiated a shift in focus. This is reflected in Hägg et al’s Cinderella hypothesis (9), which states that the smallest motor units of muscles are first and continuously active during sustained low-force work tasks, and therefore particularly susceptible to energy deficit, metabolic overload and elevated intramuscular pressure, leading to MSD (9).

Subsequently, models expanded to incorporate psychosocial and organizational work factors in the development of MSD. Bongers et al (10) developed a framework explaining how physical and psychosocial work factors interact in the development of MSD, which was further empirically supported in an extensive review by Bernard et al (11) and a reference book for effective prevention by Hagberg et al (12). Westgaard & Winkel (13) further conceptualized how organizational and contextual factors interact with the physical and psychosocial work factors to cause MSD.

Macdonald & Oakman (14) later advocated that more effective prevention of work-related MSD required a more holistic participatory system-based risk assessment and workplace procedures on all types of work-related hazard (also psychosocial) for MSD. They also developed digital guides for participative workplace risk factor identification and management (the APHIRM framework) (15). Recently, van der Beek et al (16) outlined a framework guiding the steps in workplace interventions to prevent MSD.

## Limitations of current conceptual models

As highlighted by Kuijer and colleagues in their review of work-related MSD research over the last 50 years, existing models share a common core logic: prevent work-related MSD by reducing workplace risk factors (17). This approach mirrors the logic in other domains of occupational health, such as chemical and biological occupational safety and health (OHS), treating work factors as inherent health hazards requiring elimination or reduction. This logic is exemplified by the “Vision Zero” approach (18) outlining the idea that work-related injuries and diseases are preventable through elimination of work hazards, and the Hierarchy of Controls Model (19) which guides the elimination, substitution, or engineering control of workplace hazards. These models and core logic have also expanded from their domains to other occupational health domains, including MSD prevention (20–22).

Beyond risk reduction approaches, some models in occupational health and ergonomics emphasize achieving optimal ’fit’ between workers and their work environment (14, 23). This worker–environment fit perspective focuses on matching job demands to worker capabilities and preferences rather than solely eliminating hazards (14, 23). While these fit-based approaches represent an important conceptual advance beyond pure hazard elimination, they are primarily applied to job design and crafting rather than systematically incorporated into work-related MSD prevention frameworks that promote and sustain health over time (14).

This idea of optimizing worker–environment fit aligns with the definition of ergonomics as *“the scientific discipline concerned with the understanding of interactions among humans and other elements of a system, [...] to optimize human well-being and overall system performance”* (24). The goals of the International Labour Organization (ILO) and the International Ergonomics Association (IEA) include improving worker wellbeing, occupational safety and health, and the sustainability of workers and of work systems, ie, extending beyond hazard elimination to encompass optimal worker-system fit (25). However, despite this broader conceptualization within ergonomics theory, practical MSD prevention interventions have remained largely anchored in the risk reduction paradigm.

This focus on hazard reduction and worker–environment fit contrasts with the recognition that workplaces represent big untapped potential for promoting health. As highlighted in a recent review, workers spend substantial portions of their waking hours at work, creating opportunities to influence public health through workplace-based health promotion (26). However, health promotion is traditionally considered as “something else” than work, ie, activities done besides the core work tasks. Further, despite the growing recognition of workplace health promotion, MSD prevention remains limited to the traditional risk reduction logic. An alternative is the ’total worker health’ approach that aims to integrate workplace hazard reduction with health promotion (27).

Importantly, the worker–environment fit approaches differ conceptually from risk reduction models, focusing on optimization and job crafting rather than hazard elimination. However, in the context of MSD prevention, even fit-based interventions have typically aimed to reduce hazards at work to prevent harm rather than actively promote work-related MSH.

We believe that the “workplace risk reduction logic” and fit-based approaches have merit and value in workplace prevention but also limitations when applied to MSD.

Unlike the exclusively harmful occupational exposures in other occupational health domains (eg, asbestos and carcinogenic materials), work factors relevant to MSD can also have positive influences on the musculoskeletal system. This is particularly evident with physical work factors like standing, walking, lifting, arm elevation and forward bending that are also deliberately practiced in fitness and rehabilitation settings to improve musculoskeletal function and well-being (28, 29). This can also hold for psychosocial work factors that might be beneficial for musculoskeletal well-being, such as emotionally (eg, caregiving for family) and cognitively (eg, a game of chess) demanding leisure tasks. We see it as a limitation that current influential conceptual models of work-related MSD, whether focused on risk reduction or worker–environment fit, do not capture the idea that physical and psychosocial work factors not only prevent harm or achieve fit but can have a beneficial influence on health over time.

Finally, until validated instruments to measure relevant factors and outcomes has been developed, it will be difficult to determine the value of the new conceptual approach. Such instruments are crucial for future research, but instrument development should be informed by the new approach in order to incorporate measures that are needed to evaluate the key measure and outcomes.

## Theoretical underpinnings of work’s positive health effects

The idea that a focus on health-promoting work factors adds value to a risk-reduction approach for preventing disease has been proposed and is, thus, not novel. The most influential conceptual model expanding the understanding of health beyond the mere absence of disease is arguably Antonovsky’s Salutogenesis Model (30). A central premise of this model is that health promotion is not simply the reverse of disease prevention. Instead, health is conceptualized as a continuum ranging from well-being to disease. Consequently, interventions should not only focus on reducing risk factors for illness but also on strengthening factors that actively promote health.

Occupational health models have further outlined how work factors can have both harmful and beneficial health effects. One of them is Warr’s Vitamin Model, proposing that certain psychosocial work factors (eg, job demands or autonomy) influence health in a non-linear manner, similar to the physiological effects of various vitamins (31). The model outlines that, for work factors having a curvilinear (inverted U-shaped) health effect, both excess and deficiency of exposures can be detrimental for health (31). A more recent conceptual model – the Goldilocks Work Paradigm – further operationalizes how physical and psychosocial work factors can influence health according to the described just right concept (32, 33). The paradigm proposes that work can promote workers’ health by designing physical and psychosocial work factors to be just right, ie, neither too much nor too little. Through this lens, the health effects of a physical or psychosocial work factor depend on the dose, time-pattern, combinations with other (work and non-work) factors and context. Pilot and feasibility workplace interventions based on the ’just right’ principles have shown promising effectiveness of the just right concept in several occupations (34). One cluster-randomized controlled trial among childcare workers found positive effects on worker energy at work and need for recovery, and most of the workers in the intervention group were highly satisfied with the intervention (34). However, two other trials based on this concept did not find positive effects (34). Although the evidence for the effectiveness of the ’just right’ concept is limited, the current research indicates that it has potential to be attractive to workplaces and effectiveness. In sum, we argue that these existing ideas and models offer a theoretical basis for understanding how an emphasis on promoting work-related MSH adds value to existing approaches that focus primarily on preventing MSD by reducing workplace risk factors.

## The need for a new approach to work-related musculoskeletal health

As described by Antonovsky (30), the idea that factors can have both harmful and beneficial health effects requires health-related outcome measures that reflect both the better and the worse. However, this does not align with how MSD is typically conceptualized and measured (eg, pain intensity from none (score of 0) to worst imaginary (score of 10) (35). We believe that the World Health Organization (WHO)’s definition of health as *“a state of complete physical, mental and social well-being and not merely the absence of disease or infirmity”* aligns well with this need for an instrument to measure both improvements and worsening of work-related MSH (36).

While Kuijer et al (36) recently proposed the term “work-related MSH,” we are not aware of a definition of work-related MSH that aligns with the WHO’s definition of health. We believe this hinders progress in work-related MSD research and workplace practice. To address this, we propose a new definition of work-related MSH along with a corresponding conceptual model.

## Definition of work-related musculoskeletal health

Current research and workplace prevention strategies predominantly focus on identifying and reducing work factors that can cause MSD. While this risk-reduction approach has merit, we believe it fails to capture the positive dimensions of work-related MSH, such as well-being derived during and from work. The WHO describes MSH as “*the performance of the locomotor system, comprising intact muscles, bones, joints and adjacent connective tissues*” (37). However, the organization has not provided a definition of work-related MSH. The term “MSH at the workplace” is not new (38), nevertheless we have not found a definition of it. Without a clear definition of work-related MSH, the field remains constrained to reduction of risk factors for MSD rather than enabling a comprehensive approach including how work can improve MSH.

Therefore, we propose the following definition: *“Work-related MSH is a state of physical, mental, and social well-being of the locomotor (musculoskeletal) system in relation to work.”* This definition represents a fundamental shift from avoiding MSD to optimizing how work can enhance musculoskeletal well-being. In this context of work-related MSH, physical, mental, and social well-being entails “the ability to perform work”, the “perceptions and emotions in doing work”, the “capacity to recover from and adapt to work”, the “ability to sustain to work” and the “beneficial influence from work” on the musculoskeletal system. Moreover, our understanding of the musculoskeletal system goes beyond how WHO describes the locomotor system (37), also capturing the psychosocial (eg, sensory, perceptual, cognitive) aspects. Accordingly, our definition of work-related MSH aligns with the bio-psycho-social theory capturing both physical and psychosocial aspects of work (39).

We operationalize work-related MSH as the ability of the worker to perform work tasks in a sustainable manner. Moreover, the definition captures the ability of the musculoskeletal system to perform, adapt to, recover from and sustain a variety of physical and psychosocial challenging work tasks. This encompasses traditional “work physiological” dimensions, such as strength, flexibility, endurance, and recovery capability to perform physically challenging work tasks. It also captures psychosocial aspects of well-being at work, including perceptions such as meaning, vitality, confidence, and motivation to engage in a variety of work tasks.

Our aim in proposing this definition is to establish a new foundation for understanding, research and workplace interventions and practice on work-related MSH. Advantages of the term work-related MSH include that it is:

• *Inclusive for all.* Unlike terms such as “MSD” or “pain,” which primarily emphasize workers at elevated risk or those already affected, work-related MSH offers a more inclusive perspective. This broader scope may support both early preventive efforts and the promotion of sustainable MSH for the entire workforce.

• *Better aligned with public health paradigms*. The term MSH reflects a more holistic understanding of health, consistent with the WHO’s definition. By adopting this terminology, we believe collaboration between occupational health and safety (OHS) and public health researchers and practitioners can be strengthened.

• *More engaging*. Framing the concept more in terms of MSH than MSD may foster greater engagement and motivation among workplaces, managers, employees and OHS stakeholders and policy-makers. Since sustainable work-related MSH requires both prevention and promotion, it might be more attractive for, and balanced between, employers and employees.

• *Has greater appeal to policy-makers and strategic stakeholders.* Policy-makers and system-level actors can be more responsive to concepts that emphasize sustainability, inclusion, and positive development than avoidance of MSD.

• *Encourages development of new instruments, tools and indicators for research and practice*. The new definition of work-related MSH requires development and use of new instruments for measurement of work-related MSH and implementation of workplace interventions to promote work-related MSH.

## The CoWork MSH Model

The CoWork (Copenhagen Work-related) MSH model provides a framework to support understanding how work factors can promote work-related MSH. The model is visualized in figure 1. Developed through iterative stakeholder engagement, it aims to provide OHS policy makers, stakeholders, practitioners, researchers and workplaces with a terminology, conceptual understanding, guide for translation to workplace practice, and potential benefits in shifting focus from reducing risks for MSD to promotion of work-related MSH.

**Figure:**
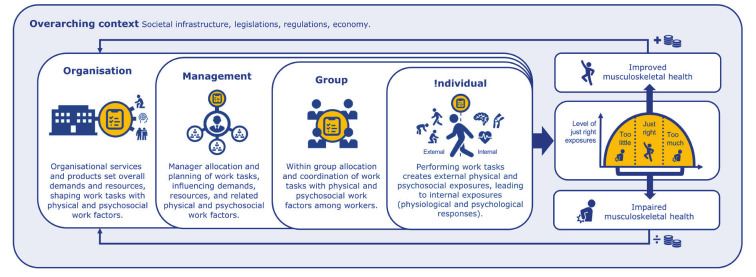
**Figure 1.** Visualization of the CoWork (Copenhagen Work-related) musculoskeletal health (MSH) model developed through iterative stakeholder engagement and feedback. The reversed IGLO model, the Organizational (O), Management (M), Group (G), Individual (!) (OMG!) workplace framework conceptualizes how work factors influence worker health within the multi-level structure of workplaces. Work tasks are the core building bricks of the model; leading to the physical and psychosocial work factors transport downwards through the organizational levels of the workplace. The physical and psychosocial work factors origin from the work tasks (illustrated by yellow symbols) performed by the workplace to fulfill its overall obligation, function and role in society. Factors in the Overarching context, such as societal regulations, infrastructure and economy have a strong influence on the overall demands and resources of the workplace. Factors such as the physical and social environment and health behaviors outside the workplace also play a role for MSH of the workers. At the Organizational (O) level, the upper-level structures, procedures, requirements and resources for producing the services or goods are set. Thus, the Organizational level has a large influence on the overall demands and resources for performing the work tasks leading to the physical and psychosocial work factors. At the Management (M) level, the more specific allocation, planning, demands and resources of the work tasks (often to groups) are determined. At the Group (G) level, teams allocate work tasks and collaborate according to the given plans, demands and resources set by the higher organizational levels. At the Individual (!) level, when the worker performs a work task with certain demands and resources, it leads to physical and psychosocial work exposures. These work factors are often called “external” physical and psychosocial exposures, such as ergonomic postures, work pace and social interactions. These external exposures further lead to physiological and psychological responses (called “internal” exposures). These internal exposures depend on the doses, time patterns, combinations with other (work and non-work) factors and context. How the internal exposures influence work-related MSH depend on how far they deviate from being just right. If they are within just right, they improve work-related MSH. While if they are “too much” or “too little” they can have a harmful influence on work-related MSH. While improvements in work-related MSH is economically beneficial for organizations, impairments of MSH leads to economic costs.

## Naming of the model

The name “CoWork” MSH model was deliberately chosen to highlight core aspects of the model. First, it highlights “Work” as the central element of the model, underscoring the importance of work factors for MSH. Further, it reinforces that “Work” is not only the context challenging MSH, but also where to promote it. Second, the prefix “Co” signals collaboration and co-creation, emphasizing the participative approach between researchers, practitioners, OHS stakeholders and policy makers, employers and employees. Third, it acknowledges the location where the model was commissioned and developed (i.e., Copenhagen). Beyond these aspects, “Co” also conveys that promotion of work-related MSH is a shared responsibility that requires collective action across the organizational levels of workplaces and beyond. It also underlines the aim to bridge OHS research and practice, highlighting that the CoWork MSH model aims to be both scientifically grounded and practically applicable.

## Model development and rationale

The development of the CoWork MSH model was initiated in response to growing concerns from Danish policymakers and social partners about the high and growing burden of work-related MSD in Denmark. Despite funding and implementation of initiatives, policies, and preventive strategies in Denmark, the prevalence and burden of MSD have remained largely unchanged or increased (40). This led to agreement among OHS stakeholders on the need for new knowledge and approaches to better understand and prevent MSD in the workplace.

In 2022, as part of the Danish Working Environment Agreement, the Working Environment Inspection Authority asked the National Research Centre for the Working Environment (NFA) to provide an overview of research-based knowledge on prevention of work-related MSD. Our (coauthors on this paper) collaboration and dialogue with colleagues at the Danish Working Environment Inspection Authority and the social partners revealed that existing models (such as the bio-psycho-social theory) were difficult to communicate and translate to workplaces. Thus, we agreed on the need to develop a new model to bridge this gap.

Close stakeholder engagement and dialogue characterized the development process of the model. The authors performed a structured literature review of work factors associated with MSD and related workplace interventions, forming a fundament for development of the model. We presented an overview of the literature review to the stakeholders in a Danish report (41), giving an updated overview of the existing research, knowledge and recommendations on work-related MSD. Subsequently, we consulted Danish social partners, OHS policymakers and practitioners across several iterations to facilitate comprehension of the model and making it engaging, and practically applicable for workplaces. Their suggestions and feedback were instrumental in shaping the conceptualization and visualization of the model (figure 1).

## Identified barriers and model purpose

This process revealed that a key barrier to workplace prevention is the absence of conceptual models that effectively bridge theoretical foundations with organizational structures and everyday practice. Without such a model, stakeholders (eg, employers, employees, and OHS practitioners) struggle to form a shared understanding of how work influences MSD, making it difficult to initiate appropriate interventions. Existing models of work-related MSD, which largely focus on risk factors, may also be less motivating for stakeholders and workplaces seeking actionable guidance. Consequently, OHS stakeholders expressed the need for a new conceptual model that moves beyond risk reduction and emphasizes the promotion of work-related MSH.

They noted that this shift—from preventing MSD to promoting MSH—requires new terminology, a clarified conceptual basis, guidance on translating concepts into workplace practice, and a clear articulation of potential benefits (eg, economic) for policymakers, practitioners, researchers, and workplaces. In response, the proposed model introduces a new definition of work-related MSH and five key elements: (i) a health-oriented approach, (ii) “just-right” conceptual thinking about work factors, (iii) the Organizational, Management, Group, !ndividual (OMG!) workplace framework, (iv) a workplace intervention perspective, and (v) an occupational health economics approach.

## Model overview and key elements

We designed the CoWork MSH model not just as a framework for research but also to promote engagement, dialogue, shared understanding, and joint action by all relevant OHS stakeholders on work-related MSH. Based on the described iterative process between the coauthors and the mentioned OHS stakeholders, the model consists of five key elements aligned with the proposed definition of work-related MSH.

*Health oriented approach.* As already described, the model reframes the focus from preventing MSD by reducing work-related risk factors to promoting and sustaining work-related MSH.

*Just right concept.* The model is based on the "just right" conceptual thinking of work and health. This means that physical and psychosocial work factors are not inherently harmful, but each can have beneficial and harmful impact on work-related MSH. This builds on the Vitamin Model and the Goldilocks Work paradigm (31, 33).

*The OMG! workplace framework.* To better align the model with workplace structures and practice, the CoWork MSH model builds on the well-established individual–group–leader–organization (IGLO) workplace framework (42, 43). IGLO is a multilevel model describing how work factors are shaped by—and can be targeted through—four organizational levels. It also incorporates the broader contextual environment surrounding the workplace, including societal regulations, policies, and economic conditions (42, 43).

During consultations, OHS stakeholders recommended reversing the IGLO sequence so that the organization, rather than the individual, appears first. Their rationale was that this ordering better reflects how workplaces function and how work factors cascade down through organizational hierarchies. Following their input, we refer to the reversed framework as the OMG! (organizational–management–group–individual) workplace framework. To support recognition and ease of communication, the individual level is denoted with an exclamation point (!), a stylized representation of the letter *i*.

*Workplace MSH intervention perspective.* As mentioned above, we consider the OMG! workplace framework and the Research Framework for the Development and Implementation of Interventions Preventing MSD (16, 42) to form a useful structure and guide to workplace interventions on work-related MSH. Because of the complexity of workplace interventions, we suggest to use the UK Medical Research Council (MRC) framework for designing, implementing and evaluating complex interventions (44). In particular, we see the systems thinking, stakeholder engagement, context adaptation and iterative development and evaluation to be valuable features of the MRC framework to base the workplace interventions to promote work-related MSH on. Moreover, already established recommendations to succeed with the design, implementation, effectiveness and evaluation of workplace interventions also apply for the CoWork MSH model (45). Examples include a participatory approach of all organizational levels, using a system approach, and a structural intervention thinking (46–48).

Compared to workplace interventions to identify and reduce workplace risk factors to prevent MSD, workplace interventions to promote MSH require a different program logic, intervention components, implementation strategies and evaluation. Because the Goldilocks Work paradigm (32, 33) shares the same "just right" concept of work factors and health, we recommend to design, implement and evaluate CoWork MSH workplace interventions according to the Goldilocks Work paradigm (32, 33).

*Occupational health economics approach.* The fifth key element of the CoWork MSH model integrates occupational health economics thinking in the concept. While economic incentives and perspectives are critical drivers of organizational decision-making, we think they are overlooked in existing models for work-related MSD. Naturally, workplaces function primarily as economic entities by producing goods and delivering services rather than promoting worker health. Consequently, economic incentives can be essential for supporting management engagement, sustained investment, and long-term implementation of workplace interventions (49). Therefore, the CoWork MSH model takes occupational health economics beyond economic evaluation of workplace interventions. Accordingly, the design and implementation of workplace interventions to promote MSH should aim for optimizing employer cost-benefit, cost-effectiveness and return on investment is a core principle. Another economic principle of the CoWork MSH model is to systematically and empirically develop and communicate business cases for workplace interventions to promote work-related MSH. The practical importance of this economic rationale became evident in our collaboration with Copenhagen municipality, which committed to substantial long-term investment in workplace MSD prevention across 300+ childcare institutions after observing positive return-on-investment (50, 51). This example illustrates how research-based economic business cases can drive scale-up, organizational adoption and investments in workplace interventions.

## Visualization of the CoWork MSH model

The CoWork MSH model is illustrated in figure 1. As seen, the OMG! framework provides a scaffold for the model. Work tasks are the core building bricks of the model, leading to the physical and psychosocial work factors transported downwards through the organizational levels of the workplace, until they finally influence workers' work-related MSH.

The physical and psychosocial work factors origins from the work tasks performed by the workplace (organization) to fulfill its overall obligation, function and role in society (the so-called “overarching context”). Factors in the overarching context – such as societal regulations, infrastructure and economy – have a strong influence on the overall demands and resources for producing services or goods by the workplace (43). In addition, factors such as the physical and social environment and health behaviors outside the workplace also play a role for workers' MSH.

At the organizational level, the upper-level structures, prioritizations, procedures, requirements and resources for producing the services or goods are set. Thus, this level has a large influence on the overall demands (eg, productivity and quality requirements, staffing) and resources (eg, time, equipment) for performing the work tasks leading to the physical and psychosocial work factors influencing workers' MSH.

At the management level, the more specific allocation, planning, demands and resources of the work tasks (often to groups) are determined. Thus, this level highly influences work factors of importance for MSH – such as level of job control and social support from management, work pressure and available ergonomic tools. Moreover, this level often has a big say on the design of the production (eg, job rotation arrangements) influencing the physical and psychosocial work factors of the workers.

At the group level, teams allocate work tasks and collaborate according to the given plans, demands and resources set by the higher organizational levels. This level often has a strong influence on who performs which work tasks, and thereby on the physical and psychosocial work factors influencing workers MSH. The MSH-related culture (eg, shared perceptions, problem solving strategies and skills, and communication in a group) is also formed at this level (52).

At the individual level, when the worker performs a work task with certain demands and resources, it leads to physical and psychosocial work exposures. These work factors are often called “external” physical and psychosocial exposures, such as ergonomic postures, work pace and social interactions. These external exposures further lead to physiological and psychological responses (called “internal” exposures) influencing the worker's MSH. These internal exposures depend on the dose, time pattern, combinations with other (work and non-work) factors and context.

In line with Warr’s Vitamin Model and the Goldilocks Work paradigm, how the internal work factors influence work-related MSH depend on how far they deviate from the “just right” principle. If they are within the acceptable range, they have a beneficial effect on work-related MSH. While if they are “too much” or “too little”, they can have a harmful influence (31–33).

## Future directions for research, implementation, and policy

The CoWork MSH model offers a new conceptual lens for promoting work-related MSH in occupational settings, going beyond prevention to improvement of MSD. Overall, the novelty of each main element of the model can be debated. However, we are not aware of any existing model that has integrated the model's main elements to address work-related MSD. More importantly, this integration enables a paradigm shift from disorder prevention to health promotion that existing MSD frameworks – whether focused on risk reduction, person-environment fit, or systems thinking – do not achieve. While each component draws on established theory, their systematic integration creates an actionable framework that stakeholders found absent from existing models, evidenced by the specific request from Danish social partners for this new approach despite the availability of established MSD frameworks. However, to realize its full potential, the model needs to be translated into practical communication materials and intervention tools for the workplace, tested through empirical research, and systematically brought into practice. Moreover, the request and development of the model has been limited to Denmark, and might not be directly applicable beyond the Nordic welfare societies. This is a limitation of the proposed model, which means that the model might need translation and tailoring to other societies. We outline the following four core areas of future research.

*Developing instruments to measure work-related MSH.* Existing measurement instruments focus predominantly on the negative outcomes of MSD, such as pain. These instruments are therefore useful for evaluating interventions or policies aimed at preventing MSD but not for promoting work-related MSH. A first research step is therefore to develop and validate measurement instruments that operationalize the proposed definition of work-related MSH (30). This step is an urgent prerequisite for successful implementation. Until validated instruments to measure relevant factors and outcomes are developed, it will be difficult to implement and determine the value of the new conceptual approach. Thus, development of instruments are essential for researchers to be able to adopt and evaluate this conceptual approach. The new approach should inform the development of these instruments in order to incorporate measures needed to evaluate the key implementation measures and outcomes.

*Building the empirical research foundation.* To support the CoWork MSH model as a research-based framework, there is a need for research that explores how various doses, time patterns, combinations with other (work and non-work) factors, and contexts are associated with work-related MSH. Much of the current research focuses on establishing dose–response thresholds to reduce risk for MSD, but the "just right" conceptual approach embedded in the CoWork MSH model calls for another perspective. Future studies should particularly explore potential non-linear or U-shaped relationships between various physical and psychosocial work factors and work-related MSH.

*Systematically designing and evaluating interventions.* Interventions according to the CoWork MSH model should follow the established recommendations to succeed with the design, implementation and evaluation of workplace interventions (45), the Research Framework for the Development and Implementation of Interventions Preventing MSD framework (16), the MRC framework (44), and the Goldilocks Work paradigm (32, 33). We encourage researchers to systematically develop, implement and evaluate workplace interventions of various designs to promote work-related MSH.

*Systematic evaluation of societal up- and downstream impact.* To fully capture the relevance and societal value of the CoWork MSH model, both its upstream and downstream societal impacts should be systematically evaluated. Societal impact from research refers to non-academic changes that can be traced back to research, such as the introduction and adoption of the CoWork MSH model. Upstream use of the model may influence policy by engaging and informing decision-makers, thereby contributing to changes in public policies, collaborative agreements, regulatory practices, or public debates. Downstream use of the model may affect workplace practices by shaping how OHS consultants advise workplaces, and how work is organized, planned and performed to improve MSH. Such intermediate outcomes should be assessed by identifying specific impact pathways and associated indicators linked to the model’s use. Widespread application of the model is expected to produce outcomes that ultimately lead to the desired end-results, such as improved work-related MSH, economic benefits, reduced MSH-related sickness absence, and increased ability, motivation and possibility to stay long at the labor market. Because such end-results are difficult to attribute directly to the model’s introduction and dissemination, careful evaluation planning of the upwards and downwards societal impact of the CoWork MSH model is needed.

## Concluding remarks

Based on extensive dialogue with key OHS stakeholders in Denmark, we have proposed a new definition of work-related MSH and developed the CoWork MSH model, which shifts the focus from merely reducing risks for work-related MSD to actively promoting work-related MSH. We hope that this new definition and conceptual model will encourage a more ambitious and comprehensive approach to how work-related MSH is understood, communicated, researched, and advanced in workplaces.

We believe the model provides a foundation for the systematic development and evaluation of measurement instruments, workplace intervention designs and tools, as well as communication and implementation strategies. In line with its name, the CoWork MSH model aims to foster collaboration and co-creation among OHS stakeholders, policymakers, practitioners, employers, employees, and researchers to strengthen work-related MSH. Ultimately, we expect the model to engage policymakers and stakeholders, stimulate new thinking and research, inspire innovative workplace interventions, and contribute to improving MSH in practice.

## References

[1] Cieza A, Causey K, Kamenov K, Hanson SW, Chatterji S, Vos T. Global estimates of the need for rehabilitation based on the Global Burden of Disease study 2019: a systematic analysis for the Global Burden of Disease Study 2019. Lancet 2021 Dec;396(10267):2006–17. 10.1016/S0140-6736(20)32340-0 33275908 PMC7811204

[2] Qiu K, Wang C, Mo X, Yang G, Huang L, Wu Y et al. The global macroeconomic burden of musculoskeletal disorders. Int J Surg 2025 Nov;111(11):7857–66. 10.1097/JS9.0000000000003072 40694023 PMC12626537

[3] Liu M, Rong J, An X, Li Y, Min Y, Yuan G et al. Global, regional, and national burden of musculoskeletal disorders, 1990-2021: an analysis of the global burden of disease study 2021 and forecast to 2035. Front Public Health 2025 Aug;13:1562701. 10.3389/fpubh.2025.1562701 40823234 PMC12354483

[4] Yazdani A, Wells R. Barriers for implementation of successful change to prevent musculoskeletal disorders and how to systematically address them. Appl Ergon 2018 Nov;73:122–40. 10.1016/j.apergo.2018.05.004 30098627

[5] Ashcraft LE, Goodrich DE, Hero J, Phares A, Bachrach RL, Quinn DA et al. A systematic review of experimentally tested implementation strategies across health and human service settings: evidence from 2010-2022. Implement Sci 2024 Jun;19(1):43. 10.1186/s13012-024-01369-5 38915102 PMC11194895

[6] Anderson CK, Chaffin DB, Herrin GD, Matthews LS. A biomechanical model of the lumbosacral joint during lifting activities. J Biomech 1985;18(8):571–84. 10.1016/0021-9290(85)90012-0 4055812

[7] Armstrong TJ, Radwin RG, Hansen DJ, Kennedy KW. Repetitive trauma disorders: job evaluation and design. Hum Factors 1986 Jun;28(3):325–36. 10.1177/001872088602800308 3770763

[8] Jørgensen K. Permissible loads based on energy expenditure measurements. Ergonomics 1985 Jan;28(1):365–9. 10.1080/00140138508963145 3996374

[9] Hagg G AP, Hobart DJ, Danhoff JV Static work loads and occupational myalgia—a new explanation model. Electromyographical kinesiology. 1991;141–4.

[10] Bongers PM, de Winter CR, Kompier MA, Hildebrandt VH. Psychosocial factors at work and musculoskeletal disease. Scand J Work Environ Health 1993 Oct;19(5):297–312. 10.5271/sjweh.1470 8296178

[11] Bernard BP, Putz-Anderson V. Musculoskeletal disorders and workplace factors: a critical review of epidemiologic evidence for work-related musculoskeletal disorders of the neck, upper extremity, and low back: Atlanta, Ga.: U.S. Dept. of Health and Human Services, Public Health Service, Centers for Disease Control and Prevention, National Institute for Occupational Safety and Health; Cincinnati, OH: National Institute for Occupational Safety and Health, Publications Dissemination distributor; Springfield, Va.: National Technical Information Service distributor; 1997.

[12] Hagberg M, Silverstein B, Wells R, Smith MJ, Hendrick HW, Carayon P et al. Work Related Musculoskeletal Disorders (WMSD): A Reference Book for Prevention. First Edition ed: Taylor & Francis; 1985.

[13] Westgaard RH, Winkel J. Guidelines for occupational musculoskeletal load as a basis for intervention: a critical review. Appl Ergon 1996 Apr;27(2):79–88. 10.1016/0003-6870(95)00062-3 15677047

[14] Macdonald W, Oakman J. Requirements for more effective prevention of work-related musculoskeletal disorders. BMC Musculoskelet Disord 2015 Oct;16:293. 10.1186/s12891-015-0750-8 26466897 PMC4606837

[15] Oakman J, Macdonald W. The APHIRM toolkit: an evidence-based system for workplace MSD risk management. BMC Musculoskelet Disord 2019 Oct;20(1):504. 10.1186/s12891-019-2828-1 31666054 PMC6822468

[16] van der Beek AJ, Dennerlein JT, Huysmans MA, Mathiassen SE, Burdorf A, van Mechelen W et al. A research framework for the development and implementation of interventions preventing work-related musculoskeletal disorders. Scand J Work Environ Health 2017 Nov;43(6):526–39. PubMed 10.5271/sjweh.3671 28945263

[17] Kuijer PP, van der Wilk S, Evanoff B, Viikari-Juntura E, Coenen P. What have we learned about risk assessment and interventions to prevent work-related musculoskeletal disorders and support work participation? Scand J Work Environ Health 2024 Jul;50(5):317–28. 10.5271/sjweh.4172 38810168 PMC11214778

[18] Belin MÅ. Johansson, Roger, Lindberg, Johan, Tingvall, Claes, editor The Vision Zero and its Consequences. The 4th international conference on Safety and the Environment; 1997; Tel Aviv, Israel

[19] de Castro AB. ‘Hierarchy of controls’. Am J Nurs 2003 Dec;103(12):104. 10.1097/00000446-200312000-00030 14702575

[20] Jilcha K. Vision Zero for industrial workplace safety innovative model development for metal manufacturing industry. Heliyon 2023 Nov;9(11):e21504. 10.1016/j.heliyon.2023.e21504 38027721 PMC10661090

[21] Davies K, Weale V, Oakman J. A Work Systems Hierarchy of Controls: Analysis of Risk Controls Developed by Paramedics. Am J Ind Med 2025 Aug;68(8):698–710. 10.1002/ajim.23741 40495295 PMC12242110

[22] Kjærgaard A, Rudolf EM, Palmqvist J, Jakobsen ME, Ajslev JZ. The Psychosocial Hierarchy of Controls: Effectively Reducing Psychosocial Hazards at Work. Am J Ind Med 2025 Mar;68(3):250–63. 10.1002/ajim.23694 39707860 PMC11834945

[23] Wilson JR. Fundamentals of systems ergonomics/human factors. Appl Ergon 2014 Jan;45(1):5–13. 10.1016/j.apergo.2013.03.021 23684119

[24] Association IE. What Is Ergonomics (HFE)? Available from: https://iea.cc/about/what-is-ergonomics/

[25] (IEA) IEA. (ILO) ILO. Principles and Guidelines for Human Factors/Ergonomics (HF/E) Design and Management of Work Systems 2021.

[26] Andersen LL. Health Promotion and Chronic Disease Prevention at the Workplace. Annu Rev Public Health 2024 May;45(1):337–57. 10.1146/annurev-publhealth-060222-035619 37788631

[27] Lee MP, Richards R, Chang CC, Chosewood LC, Schill AL; NIOSH Office for Total Worker Health. Fundamentals of total worker health approaches: essential elements for advancing worker safety, health, and well-being. DHHS (NIOSH) Publication No. 2017–112;2016: Department of Health and Human Services, Centers for Disease Control and Prevention, National Institute for Occupa-tional Safety and Health; 2016.

[28] Straker L, Holtermann A, Lee IM, van der Beek AJ, Stamatakis E. Privileging the privileged: the public health focus on leisure time physical activity has contributed to widening socioeconomic inequalities in health. Br J Sports Med 2020 Oct;bjsports-2020-103356. 10.1136/bjsports-2020-103356 33004406

[29] Comachio J, Beckenkamp PR, Ho EK, Shaheed CA, Stamatakis E, Ferreira ML et al. Benefits and harms of exercise therapy and physical activity for low back pain: an umbrella review. J Sport Health Sci 2025 Dec;14:101038. 10.1016/j.jshs.2025.101038 40180212 PMC12191304

[30] Antonovsky A. The salutogenic model as a theory to guide health promotion. Health Promot Int 1996;11(1):11–8. 10.1093/heapro/11.1.11

[31] De Jonge J, Schaufeli WB. Job characteristics and employee well-being: a test of Warr’s Vitamin Model in health care workers using structural equation modelling. J Organ Behav 1998;19:387–407. 10.1002/(SICI)1099-1379(199807)19:4<387::AID-JOB851>3.0.CO;2-9

[32] Holtermann A, Mathiassen SE, Straker L. Promoting health and physical capacity during productive work: the Goldilocks Principle. Scand J Work Environ Health 2019 Jan;45(1):90–7. 10.5271/sjweh.3754 30021029

[33] Straker L, Mathiassen SE, Holtermann A. The Goldilocks Work Paradigm: Conception, Experience, Refinement, and Future. IISE Trans Occup Ergon Hum Factors 2025 Apr;(Apr):1–13. 10.1080/24725838.2025.2491483 40256986

[34] Schmidt KG, Lerche AF, Christensen MR, Rasmussen CL, Straker L, Mathiassen SE et al. Effectiveness of a Goldilocks Work intervention in childcare workers - A cluster-randomized controlled trial. Scand J Work Environ Health 2024 Apr;50(3):197–207. 10.5271/sjweh.4145 38436676 PMC11057501

[35] Breivik H, Borchgrevink PC, Allen SM, Rosseland LA, Romundstad L, Hals EK et al. Assessment of pain. Br J Anaesth 2008 Jul;101(1):17–24. 10.1093/bja/aen103 18487245

[36] WHO. Health and Well-Being. Available from: https://www.who.int/data/gho/data/major-themes/health-and-well-being. World Health Organization.

[37] WHO. Musculoskeletal health. Available from: https://www.who.int/news-room/fact-sheets/detail/musculoskeletal-conditions. World Health Organization; 2022.

[38] Crawford JO, Berkovic D, Erwin J, Copsey SM, Davis A, Giagloglou E et al. Musculoskeletal health in the workplace. Best Pract Res Clin Rheumatol 2020 Oct;34(5):101558. PubMed 10.1016/j.berh.2020.101558 32680769

[39] Borrell-Carrió F, Suchman AL, Epstein RM. The biopsychosocial model 25 years later: principles, practice, and scientific inquiry. Ann Fam Med 2004;2(6):576–82. 10.1370/afm.245 15576544 PMC1466742

[40] Sundhedsstyrelsen. Danskernes Sundhed - Den Nationale Sundhedsprofil 2021. Available from: https://www.sst.dk/da/udgivelser/2022/Danskernes-sundhed; 2022.

[41] Jacobsen SS, Lindberg L, Andersen LL, Holtermann A. Vidensopsamling om effektiv forebyggelse af MSB og fysisk nedslidning. Det Nationale Forskningscenter for Arbejdsmiljø; 2024.

[42] Nielsen K, Yarker J, Munir F, Bültmann U. IGLOO: an integrated framework for sustainable return to work in workers with common mental disorders. Work Stress 2018;32(4):400–17. 10.1080/02678373.2018.1438536

[43] Nielsen K, Miraglia M. What works for whom in which circumstances? On the need to move beyond the ‘what works?’ question in organizational intervention research. Hum Relat 2016;70(1):40–62. 10.1177/0018726716670226

[44] Skivington K, Matthews L, Simpson SA, Craig P, Baird J, Blazeby JM et al. A new framework for developing and evaluating complex interventions: update of Medical Research Council guidance. BMJ 2021 Sep;374(2061):n2061. 10.1136/bmj.n2061 34593508 PMC8482308

[45] von Thiele Schwarz U, Nielsen K, Edwards K, Hasson H, Ipsen C, Savage C et al. How to design, implement and evaluate organizational interventions for maximum impact: the Sigtuna Principles. Eur J Work Organ Psychol 2020 Aug;30(3):415–27. 10.1080/1359432X.2020.1803960 34518756 PMC8432268

[46] Rivilis I, Van Eerd D, Cullen K, Cole DC, Irvin E, Tyson J et al. Effectiveness of participatory ergonomic interventions on health outcomes: a systematic review. Appl Ergon 2008 May;39(3):342–58. 10.1016/j.apergo.2007.08.006 17988646

[47] Whelan J, Fraser P, Bolton KA, Love P, Strugnell C, Boelsen-Robinson T et al. Combining systems thinking approaches and implementation science constructs within community-based prevention: a systematic review. Health Res Policy Syst 2023 Aug;21(1):85. 10.1186/s12961-023-01023-4 37641151 PMC10463953

[48] Aust B, Møller JL, Nordentoft M, Frydendall KB, Bengtsen E, Jensen AB et al. How effective are organizational-level interventions in improving the psychosocial work environment, health, and retention of workers? A systematic overview of systematic reviews. Scand J Work Environ Health 2023 Jul;49(5):315–29. 10.5271/sjweh.4097 37158211 PMC10713994

[49] Martinsson C, Lohela-Karlsson M, Kwak L, Bergström G, Hellman T. What incentives influence employers to engage in workplace health interventions? BMC Public Health 2016 Aug;16(1):854. 10.1186/s12889-016-3534-7 27552912 PMC4995638

[50] Mia Nyvang Stilling LL. Laura Downs Tuck, Ole Henning Sørensen, Andreas Holtermann, Charlotte Diana Nørregaard Rasmussen. Copen-SCALE: scaling-up an effective intervention to childcare institutions in Copenhagen – a stepped wedge type II hybrid effectiveness-implementation trial. A study protocol. BMC Public Health 2025. 10.1186/s12889-025-24463-9 PMC12513091 41068663

[51] Gupta N, van Dongen JM, Holtermann A, van der Beek AJ, Stevens ML, Nørregaard Rasmussen CD. Cost-Effectiveness and Return-on-Investment of a Participatory Ergonomics Intervention Among Childcare Workers: An Economic Evaluation in a Randomized Controlled Trial. J Occup Environ Med 2022 Jun;64(6):533–9. 10.1097/JOM.0000000000002510 35143453 PMC9275851

[52] Ajstrup M, Budtz CR, Nielsen KJ, Andersen DR, Andersen JH, Christiansen DH. Musculoskeletal Health Climate Is a Prognostic Determinant of Sickness Absence Among Female Eldercare Workers: A Prospective Cohort Study. J Occup Environ Med 2023 Jan;65(1):e4–9. 10.1097/JOM.0000000000002729 36240746 PMC9835667

